# Neuroprotective effects of verbascoside against Alzheimer’s disease via the relief of endoplasmic reticulum stress in Aβ-exposed U251 cells and APP/PS1 mice

**DOI:** 10.1186/s12974-020-01976-1

**Published:** 2020-10-18

**Authors:** Chunyue Wang, Xueying Cai, Ruochen Wang, Siyu Zhai, Yongfeng Zhang, Wenji Hu, Yizhi Zhang, Di Wang

**Affiliations:** 1 School of Life Sciences, Jilin University, Changchun, 130012 China; 2 Department of Neurology, The Second Hospital of Jilin University, Changchun, 130041 China

**Keywords:** Alzheimer’s disease, Verbascoside, Endoplasmic reticulum stress, Unfolded protein response, Aβ

## Abstract

**Background:**

Endoplasmic reticulum (ER) stress is involved in the progression of Alzheimer’s disease (AD). Verbascoside (VB), an active phenylethanoid glycoside that was first isolated from *Verbascum sinuatum* (the wavyleaf mullein), possesses anti-inflammatory, antioxidative, and anti-apoptotic effects. The purpose of this study was to elucidate the beneficial effects of VB in amyloid β (Aβ)_1–42_-damaged human glioma (U251) cells and in APPswe/PSEN1dE9 transgenic (APP/PS1) mice.

**Methods:**

U251 cells were co-incubated with 10 μM of Aβ_1-42_ and treated with VB. The protective effects of VB were investigated by using 3-(4,5-dimethylthiazole-2-yl)-2,5-diphenyl tetrazolium bromide assay, flow cytometry, fluorescence staining, and transmission electron microscopy. APP/PS1 transgenic mice were treated for 6 weeks with VB. Learning and memory were evaluated using a Morris water maze test. Immunohistochemistry, terminal deoxynucleotidyl transferase-mediated deoxyuridine triphosphate nick end labeling, thioflavin-S staining, and proteomics analysis were performed to study the potential neuroprotective mechanism. Enzyme-linked immunosorbent assays and western blot were performed to analyze altered protein levels of brain lysates in APP/PS1 mice and/or Aβ_1-42_-damaged U251 cells.

**Results:**

In Aβ_1-42_-damaged U251 cells, VB significantly improved cell viability, inhibited apoptosis, reduced calcium accumulation and the intracellular concentrations of reactive oxygen species, and improved the morphology of mitochondria and ER. In APP/PS1 mice, 6-week administration of VB significantly improved memory and cognition. VB inhibited apoptosis, reduced the deposition of Aβ, reduced the formation of neurofibrillary tangles formed by hyperphosphorylated tau protein, and downregulated the expression levels of 4-hydroxynonenal and mesencephalic astrocyte-derived neurotrophic factor in the brains of APP/PS1 mice. Proteomics analysis of mouse hippocampus suggested that the neuroprotective effect of VB may be related to the reduction of ER stress. This was indicated by the fact that VB inhibited the three branches of the unfolded protein response, thereby attenuating ER stress and preventing apoptosis.

**Conclusions:**

The results confirmed that VB possesses significant neuroprotective effects, which are related to the reduction of ER stress. These findings support the status of VB as a potentially effective treatment for AD and warrant further research.

## Introduction

Alzheimer’s disease (AD) is a common degenerative brain disease in the elderly [1, 2] and is clinically characterized by memory decline and cognitive dysfunction [3]. According to previous reports, approximately 13% of people aged over 65 develop AD. The quality of life of these individuals is severely reduced due to language and behavioral barriers [4]. Furthermore, patients die within an average of 10 years after their AD diagnosis [5]. The deposition of amyloid β (Aβ) and neurofibrillary tangles (NFTs) formed by hyperphosphorylated tau protein are two pathologic hallmarks of AD, which eventually lead to neuronal death and cognitive impairment [6, 7]. As the pathogenesis of AD is unclear, there is a lack of effective early prevention and treatment methods [8]. Although some treatments temporarily relieve AD symptoms, thereby improving the quality of life of patients and reducing caregiver burdens, there is currently no cure for AD, nor a treatment to halt its progression [9].

Drugs approved by the US Food and Drug Administration for AD include cholinesterase inhibitors (tacrine, rivastigmine, galantamine, and donepezil) and *N*-methyl-d-aspartate receptor antagonists (memantine) [9, 10]. However, due to its hepatotoxic and gastrointestinal side effects, the cholinesterase inhibitor tacrine was withdrawn from clinical use in 2003 [11], and this group of drugs often cause nausea, diarrhea, vomiting, and unconsciousness [12, 13]. In addition, memantine increases the risk of hypertension, confusion, and neurological diseases in the elderly [14]. An encouraging potential treatment is GV-971, a sodium oligosaccharide that is extracted from marine plants, which exhibits a good therapeutic effect in patients with mild to moderate AD by improving the intestinal microenvironment [15]. This compound was approved for AD treatment in China in 2019 (State Drugs Administration License No.: GUOYAOZHUNZI H20190031). In general, plant-derived therapies have attracted the attention of researchers screening candidate agents for various diseases due to their extensive pharmacological activities. For example, verbascoside (VB), also known as acteoside, was first isolated from the herbaceous plant *Verbascum sinuatum* (the wavyleaf mullein) in 1968 (Additional file 1: Fig. S1). VB is an active phenylethanoid glycoside and possesses significant anti-inflammatory, antioxidative, and anti-apoptotic activities, among other biological activities [16, 17] and can cross the blood-brain barrier in adult zebrafish [18]. In clinical trials, the platelet aggregation value of patients who took 100 mg/d of VB for 2 weeks was significantly reduced, without observable adverse reactions [19]. Studies of the neuroprotective effects of VB have shown that it can increase the memory capabilities of mice with d-galactose- and AlCl_3_-induced AD by reducing the expression levels of caspase 3 [20], repair scopolamine-induced memory impairment in ICR mice [21], and inhibit the aggregation of Aβ [22]. Furthermore, the anti-apoptotic effects of VB protect SH-SY5Y (neuroblastoma) cells against Aβ-induced damage [16]. However, systematic in vitro and in vivo studies have not been reported on the neuroprotective effects of VB with respect to endoplasmic reticulum (ER) stress.

Due to its role in regulating cell apoptosis, ER stress has been reported to be involved in the progression of AD [23]. It is known that the molecular chaperone immunoglobulin-binding protein (BiP) binds to misfolded or unfolded proteins and releases pressure sensors to stimulate the unfolded protein response (UPR) during ER stress [24]. If ER homeostasis cannot be recovered in time, the UPR fails to resolve these pressure signals from the ER, leading to apoptosis [5]. A feedback loop may develop, in which ER stress promotes Aβ neurotoxicity [25] and enhances tau protein phosphorylation, which further triggers the UPR in neurons, generating a vicious cycle that drives the progression of AD [26]. Astrocytes are widespread in the central nervous system [27] and have been reported to respond to the oligomerization of Aβ peptides by regulating the release of calcium ions (Ca^2+^) in the ER, which triggers ER stress and causes reactive astrogliosis, ultimately leading to the damaged neuronal signal transmission seen in AD [28]. Human glioma (U251) cells showing an effective astrogliotic response have been used as an astrocyte model to investigate the expression of glial fibrillary acidic protein and Toll-like receptors in previous studies [29, 30], and Aβ_1-42_-damaged U251 cells are commonly used as a typical in vitro AD model [31, 32].

In this study, we explored the neuroprotective effects of VB in Aβ-damaged U251 cells and APPswe/PSEN1dE9 transgenic (APP/PS1) mice. We used proteomics, western blot, and enzyme-linked immunosorbent assay (ELISA) techniques to determine whether the neuroprotective effects of VB against AD were related to the regulation of ER stress. The resulting data provide a theoretical basis for the further investigation of the neuroprotective properties of VB.

## Materials and methods

### Cell culture

U251 cells were obtained from the BeNa Culture Collection (No. BNCC337874) (Beijing, China) and cultured in Dulbecco’s modified Eagle’s medium (Gibco, Thermo Fisher Scientific, Waltham, MA, USA) supplemented with 10% fetal bovine serum (Zhejiang Tianhang Biotechnology Co., Ltd., Huzhou, China), 1% 100 μg/mL streptomycin, and 100 units/mL penicillin (Gibco, Thermo Fisher Scientific, Waltham, MA, USA) in a humidified 5% CO_2_ incubator at 37 °C.

### Cell viability assay

U251 cells were seeded into 96-well plates at a density of 8 × 10^3^ cells per well. After 12 h of incubation, cells were pretreated with VB (Cas No. 61276-17-3, purity 98.38%, Chengdu Herb-purify Co., Ltd., China) for 3 h at doses of 0.25 and 1 μM, and then co-incubated with or without 10 μM of Aβ_1-42_ (052487, Gill Biochemical Co., Ltd., Shanghai, China) at 37 °C for a further 24 h. Twenty microliters of 3-(4,5-dimethylthiazole-2-yl)-2,5-diphenyl tetrazolium bromide (5 mg/mL) was added to each well, and the cells were further incubated for 4 h at 37 °C in darkness. After removing the supernatant, 150 μL of dimethyl sulfoxide were added to dissolve crystalline formazan. The absorbance at 490 nm was measured using a microplate reader (Synergy 4, Omega Bio-tek, Inc., Norcross, GA, USA).

### Measurement of intracellular reactive oxygen species (ROS) and Ca^2+^ concentrations

As before [33], U251 cells were seeded into 6-well plates at a density of 2 × 10^5^ cells per well, pretreated with 0.25 and 1 μM of VB for 3 h, and co-incubated with or without 10 μM of Aβ_1-42_ at 37 °C for 12 h. The intracellular concentrations of ROS were measured using an ROS assay kit (E004, Nanjing Jiancheng Bioengineering Institute, Nanjing, China) according to the manufacturer’s instructions. The intracellular concentrations of Ca^2+^ were measured using Fluo-4 AM dye (F14201, Thermo Fisher Scientific, Waltham, MA, USA) according to the manufacturer’s instructions. The fluorescence intensity of cells under light excitation was observed using a fluorescent inverted microscope (CKX31, Olympus, Tokyo, Japan).

### Measurement of apoptosis and transmission electron microscopy (TEM)

The protocol was adjusted based on a previous study [3]. U251 cells were seeded into 6-well plates at a density of 2 × 10^5^ cells per well, pretreated with 0.25 μM and 1 μM of VB for 3 h, and then co-incubated with or without 10 μM of Aβ_1-42_ at 37 °C for 24 h. An Annexin V-FITC/PI Apoptosis Detection kit (Muse, Merck, Darmstadt, Germany) was used to stain the cells according to the manufacturer’s instructions, and the apoptotic rates were assessed using a Muse™ Cell Analyzer (Muse, Merck, Darmstadt, Germany).

Treated cells were collected, fixed with 4% glutaraldehyde (R20513, ShangHai YuanYe Bio-technology co., Ltd., Shanghai, China) overnight, and post-fixed in 1% osmium tetroxide at 4 °C for 2 h. After gradient dehydration using ethanol, acetone, and propylene oxide, cells were embedded in SPI-Pon 812 resin and cut into ultra-thin sections using an ultra-thin microtome (EM UC7, Leica Microsystems, Wetzlar, Germany). After staining with uranyl acetate and lead citrate, the morphology of mitochondria and ER in the cells was observed by TEM (H-7650, HITACHI, Tokyo, Japan).

### Animals and administration

The experiments complied with the Animal Research: Reporting In Vivo Experiments (ARRIVE) guidelines and were carried out in accordance with the National Institutes of Health guide for the care and use of laboratory animals. We obtained an ethical review regarding animal welfare, which was approved by the Experimental Animal Center of Jilin University (No. SY201905014). All of the mice were purchased from the Nanjing Biomedical Research Institute of Nanjing University, Jiangsu, China (SCXK [SU] 2015-0001), and raised in a room at a temperature of 23 ± 2 °C and a humidity of 40–60%, under a 12-h:12-h light:dark cycle and with free access to water and food.

**Fig. 1:**
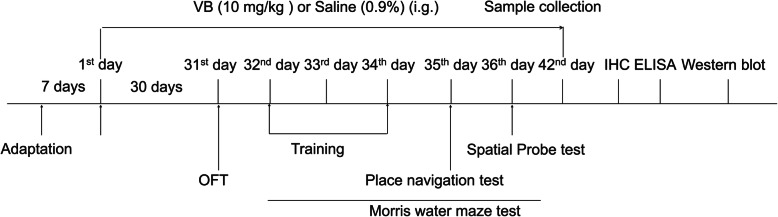
APP/PS1 mice experiment timeline

Twenty-four B6C3-Tg (APPswePSEN1dE9)/Nju double-transgenic male mice (genotype: (Appswe) T, (Psen1) T) (APP/PS1) (8 months old, 40.7–52.9 g) were randomly divided into two groups and given 0.4 mL of normal saline (*n* = 12) or 10 mg/kg of VB (*n* = 12) orally for 42 days. An additional 12 wild-type (WT) male mice (genotype: (Appswe) W, (Psen1) W) (8 months old, 41.4–51.9 g) were treated orally with 0.4 mL of normal saline each day for 42 days. Behavioral training was started after 30 days of VB administration. After the entire 42-day VB treatment period, all of the mice were euthanized via intraperitoneal injection of sodium pentobarbital (150 mg/kg). Sera and organs, comprising the brain, kidney, spleen, and liver, were quickly collected. Brain, liver, spleen, and kidney tissues of mice from each group were fixed in 4% paraformaldehyde (*n* = 3/group), and the remaining tissues were immediately stored at − 140 °C for testing (*n* = 9/group). The experimental schedule is shown in Fig. 1.

### Behavioral tests

#### Open-field test (OFT)

The experimental procedure was modified based on a previously reported approach [34]. On the 31st day, an OFT was performed of all experimental mice in each group (*n* = 12/group) (Fig. 1). The open-field device was 50 × 50 cm and divided into a central area of 25 × 25 cm and a surrounding area (ZS_ZFT;ZS DiChuang Technology Development Co., Ltd., Beijing, China). In the formal test, each mouse was placed into the open field at the same location. The trajectory of mice, their total distance moved, and the time spent in the central area were recorded by camera for 5 min and analyzed using ANY-maze^TM^ video-tracking software (Stoelting Co., Chicago, IL, USA). The OFT apparatus was cleaned before testing each mouse to ensure that no information from prior tests was present.

#### Morris water maze (MWM) test

The protocol was adjusted based on a previously reported method [35]. An MT-200 Water Labyrinth Video Tracking Analysis System (MT-200) (TECHMAN Software Co., Ltd., Chengdu, China) with a 100-cm-diameter pool was used for MWM tests of all of the experimental mice (*n* = 12/group). The circular pool was filled to a 40-cm depth with water (24 ± 2 °C) containing titanium dioxide. The water level was 0.5 cm higher than the height of the platform. From the 32nd day, the mice were trained four times per day for 3 days. During the training process, the mice were placed on the platform for 15 s and then put into the maze facing the wall at the position furthest from the platform. Mice were then allowed 60 s to find the platform. The swimming speed of each mouse and the time it took to find the platform were recorded. The mice that failed to find the platform were manually placed on the platform for 15 s. The place navigation test was executed on the 35th day. Each mouse was placed in the maze at the same location as during training. The movement trajectory, time spent searching for the platform, and swimming speed of all mice were recorded using a camera over 60 s. If a mouse did not find the platform within 60 s, its search time was recorded as 60 s. A spatial probe test was conducted on the 36th day (Fig. 1). On the same day, the platform was taken away, and the mice were again put into the maze at the same position as before. The movement trajectory of mice, the number of times the mice crossed the previous location of the platform, and the time that mice spent in the effective area were recorded using a camera over 60 s. All of the data were analyzed using Watermaze2.0 software (TECHMAN Software Co., Ltd., Chengdu, China).

### Hematoxylin and eosin (H&E) staining

As described in our previous research [36], brain, liver, spleen, and kidney tissues were fixed in 4% paraformaldehyde, embedded in paraffin, cut to a thickness of 5 μm, and stained with H&E. The sections (*n* = 3/group) were observed using an optical microscope (BX51, Olympus, Tokyo, Japan).

### Terminal deoxynucleotidyl transferase-mediated deoxyuridine triphosphate nick end labeling (TUNEL) and thioflavin-S staining

As in our previous study [37], the paraffin-embedded brain sections were soaked in xylene, treated with an ethanol gradient (100%, 90%, 80%, 70%), and incubated with terminal deoxynucleotidyl transferase and Click-iT™ Plus (Catalog Nos. C10617, Invitrogen, Thermo Fisher Scientific, Waltham, MA, USA) according to the instructions supplied by the manufacturer for TUNEL staining. For thioflavin-S staining, ethanol gradient-treated sections were stained with 0.3% thioflavin-S at 25 °C for 8 min and then washed with 50% ethanol. All of the sections (*n* = 3/group) were observed using a fluorescence microscope (BX53, Olympus, Tokyo, Japan).

### Immunohistochemical analyses

Similar to our previous protocol [33], the paraffin-embedded brain sections were soaked in xylene, treated with an ethanol gradient (95%, 85%, 75%), blocked with 3% H_2_O_2_ for 10 min, sealed with 10% goat serum (Bioss, Beijing, China) for 30 min, and incubated with primary antibodies for Aβ_1-42_, tau (phosphor S396), 4-hydroxynonenal (4-HNE), and mesencephalic astrocyte-derived neurotrophic factor (MANF) at 4 °C overnight. The sections were incubated with goat anti-rabbit IgG (H+L) (peroxidase/HRP conjugated) and subjected to 5% diaminobenzidine oxidation. After staining with DAB (Solarbio, Beijing, China), the sections (n = 3/group) were observed using an optical microscope (BX51, Olympus, Tokyo, Japan). The details of the above antibodies are presented in Table 1.

**Table 1:** Details of antibodies used in immunohistochemical and western blot

Number	Antibodies	Molecular weight	Catalog number	Dilution	Application	Company	Area
1	Aβ_1-42_		bs-0107R	1:800	Immunohistochemical	Bioss	Beijing, China
2	4-HNE		ab46545	1:200	Immunohistochemical	Abcam	Cambridge, MA, USA
3	tau (phosphor S396)		ab109390	1:4000	Immunohistochemical	Abcam	Cambridge, MA, USA
4	MANF		ab67271	1:400	Immunohistochemical	Abcam	Cambridge, MA, USA
5	MANF	20 kDa	ab67271	1:1000	Western blot	Abcam	Cambridge, MA, USA
6	BiP	78 kDa	ab21685	1:250	Western blot	Abcam	Cambridge, MA, USA
7	p-IRE1 phosphor S724	110 kDa	ab48187	1:1000	Western blot	Abcam	Cambridge, MA, USA
8	CHOP	27 kDa	ab11419	1:1000	Western blot	Abcam	Cambridge, MA, USA
9	IRE1	105 kDa	BS-8680R	1:1000	Western blot	Bioss	Beijing, China
10	XBP1	40 kDa	bs-1668R	1:1000	Western blot	Bioss	Beijing, China
	PERK	122 kDa	BSM-51385M	1:1000	Western blot	Bioss	Beijing, China
11	ATF6	75 kDa	bs-1634R	1:500	Western blot	Bioss	Beijing, China
12	ATF4	38 kDa	bs-1531R	1:5000	Western blot	Bioss	Beijing, China
13	p-PERK (Thr982)	125 kDa	DF7576	1:800	Western blot	Affinity Biosciences	Cincinnati, OH, USA
14	eIF2α	38 kDa	9722S	1:1000	Western blot	Cell Signaling Technology	Beverly, MA, USA
15	p-eIF2α (Ser51)	38 kDa	9721S	1:1000	Western blot	Cell Signaling Technology	Beverly, MA, USA
16	caspase12	38 kDa	A0217	1:1000	Western blot	ABclonal	Wuhan, China
17	GAPDH	37 kDa	E-AB-20059	1:2000	Western blot	Elabscience	Wuhan, China
18	Goat anti-mouse		E-AB-1001	1:5000	Western blot	Elabscience	Wuhan, China
19	Goat anti-rabbit		E-AB-1003	1:5000	Western blot	Elabscience	Wuhan, China

### Label-free quantification proteomics

Similar to a previously reported study [38], the brain samples (*n* = 6/group) were lysed using radio-immunoprecipitation assay (RIPA) buffer containing phenylmethanesulfonylfluoride (PMSF), stored on ice, and quantified using a Pierce™ BCA Protein Assay Kit (23225, Thermo Scientific, Waltham, MA, USA). After the protein was precipitated with pre-cooled acetone, ammonium bicarbonate (containing 1% sodium deoxycholate), tris-(2-carboxyethyl)-phosphine, iodoacetamide, and trypsin were successively added to the protein. Trifluoroacetic acid was added to remove sodium deoxycholate from the protein. After desalting, the samples were analyzed using liquid chromatography–tandem mass spectrometry (LC-MS/MS). For each sample, approximately 2 μg of peptide were isolated and analyzed with nano ultra-performance liquid chromatography (EASY-nLC1200) coupled to Q-Exactive mass spectrometry (Thermo Finnigan). Chromatographic separation was performed on a reverse-phase column (100 μm, ID × 15 cm, Reprosil-Pur 120 C18-AQ, 1.9 μm, Dr. Math). Statistical analysis was performed on the standardized quantitative results to obtain the corresponding differentially expressed proteins. Raw MS files were processed with MaxQuant (Version 1.5.6.0). The protein sequence database was downloaded from UniProt. Proteins that were significantly differentially expressed were used to perform clustering analysis and presented as a heatmap. Protein interaction analysis was performed using the STRING database. All of the above reagents were purchased from Sigma-Aldrich (St. Louis, MO, USA).

### Elisa

The brain tissue samples (*n* = 8/group) were lysed with RIPA buffer containing PMSF and stored on ice. The collected protein samples were quantified using a Pierce™ BCA Assay Kit. The concentrations of the following proteins were analyzed using the corresponding commercial ELISA kits (Jiangsu Kete Biological Technology Co., Ltd, Jiangsu, China): caspase 3 (F9179-A), caspase 12 (KT9236-A), caspase 8 (KT9229-A), insulin-degrading enzyme (IDE) (KT9252-A), ROS (KT2800-A), X-box binding protein 1 (XBP1) (KT9259-A), inositol requiring protein 1 (IRE1) (KT9244-A), activating transcription factor-6 (ATF6) (KT9235-A), MANF (KT9262-A), C/EBP-homologous protein (CHOP) (KT9231-A), pancreatic ER kinase (PERK) (KT9250-A), Aβ_1-42_ (KT9256-A), BiP (KT9240-A), eukaryotic initiation factor 2 (eIF2α) (KT9439-A), activating transcription factor-4 (ATF4) (KT9373-A), phosphorylated (p)-eIF2α(KT9422-A), p-PERK (KT9413-A), and p-IRE1 (KT9432-A). The absorbance at 450 nm was detected using a microplate reader (Synergy 4, Omega BioTek, Inc., Norcross, GA, USA).

### Western blot

U251 cells were seeded into 6-well plates at a density of 2 × 10^5^ cells per well, pretreated with 0.25 and 1 μM of VB for 3 h, and then co-incubated with or without 10 μM of Aβ_1-42_ at 37 °C for 24 h. The brain tissues collected from experimental mice and treated cells were lysed using RIPA buffer containing PMSF, stored on ice, and quantified using the Pierce™ BCA Protein Assay Kit. Samples in each group contained brain tissues from three mice.

As in our previous study [39], 40 μg of protein and 4 μL of marker (26616, Thermo scientific, Waltham, MA, USA) were separated using sodium dodecyl sulfate-polyacrylamide gel electrophoresis and then transferred to polyvinylidene fluoride membranes (0.45 μm) (GE Healthcare Life Science, Beijing, China). The loaded membranes were blocked in 5% bovine serum albumin at 4 °C for 5 h and then incubated separately with the following primary antibodies at 4 °C overnight: MANF, BiP, p-IRE1 (phosphor S724), CHOP, ATF6, IRE1, XBP1, PERK, ATF4, p-PERK (Thr982), eIF2α, p-eIF2α, caspase12, and glyceraldehyde 3-phosphate dehydrogenase (GAPDH). After washing with tris-buffered saline containing 0.1% Tween 20, the membranes were incubated separately with the following secondary antibodies: goat anti-mouse IgG (H+L) (peroxidase/HRP conjugated) and goat anti-rabbit IgG (H+L) (peroxidase/HRP conjugated). The membranes were then exposed using an electrochemiluminescence kit (Merck, Darmstadt, Germany) to obtain blots, which were visualized using an automatic chemiluminescence image analysis system (Tanon 5200, Tanon Science & Technology Co., Ltd., Shanghai, China) and quantified using Image-pro Plus 6.0 analysis software (Media Cybernetics, Bethesda, MD, USA). The details of the above antibodies are presented in Table 1.

### Statistical analysis

Data were expressed as means ± standard errors of the mean (SEM). Statistical significance was determined using one-way analysis of variance with SPSS 16.0 software (IBM Corp., Armonk, NY, USA). Post hoc analysis was performed using multiple comparisons (Dunn’s test). A *p* value < 0.05 was considered to indicate a statistically significant difference. All of the graphs were produced using GraphPad Prism 7.0 (GraphPad Software Inc., San Diego, CA, USA).

## Results

### Neuroprotective effects of VB against Aβ_1-42_-induced neurotoxicity in U251 cells

**Fig. 2:**
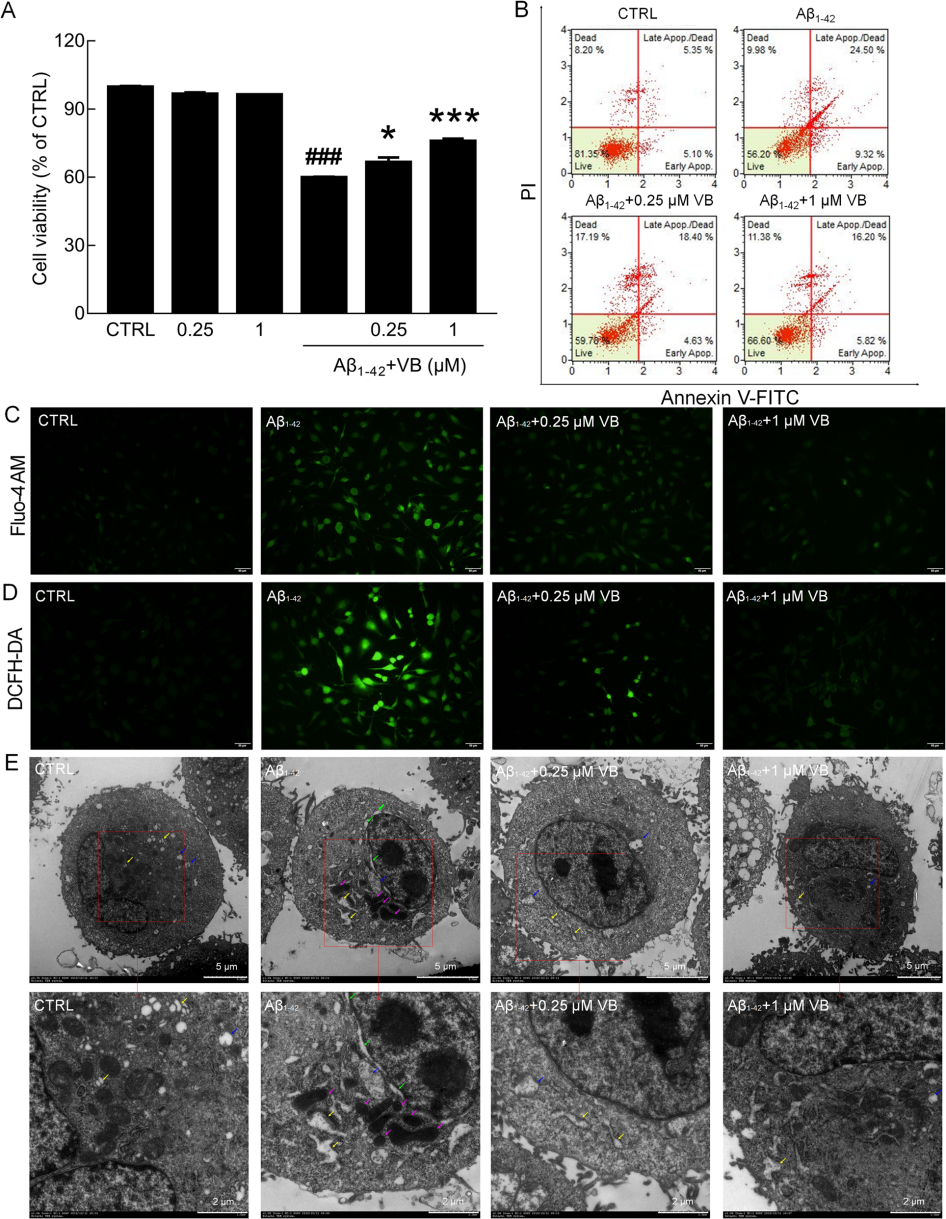
VB attenuated Aβ_1-42_-induced U251 cell apoptosis. **a** VB increased cell viability that had been reduced by Aβ_1-42_ exposure, but failed to improve cell viability alone (*n* = 6/group). The data are presented as mean ± SEM. ^###^*p* < 0.001 vs. CTRL, and *p < 0.05, ***p < 0.001 vs. Aβ_1-42_-exposed U251 cells. **b** VB reduced the apoptotic rate of Aβ_1-42_-exposed U251 cells (*n* = 4/group). **c** Fluorescence microscopy showed that VB suppressed the Ca^2+^ flux caused by Aβ_1-42_ (scale bar: 50 μm, magnification × 20) (*n* = 4/group). **d** Fluorescence microscopy showed that VB suppressed the over-accumulation of intracellular reactive oxygen species (scale bar: 50 μm, magnification × 20) (*n* = 4/group). **e** TEM analysis showed that VB improved ER and mitochondrial morphology. The expansion of ER was marked by yellow arrows, the mitochondrial cavitation was marked by blue arrows, the mitochondrial electron density increase was marked by pink arrows, and the space around the nucleus was marked by green arrows (scale bar 5 μm, magnification × 0.7 k) (scale bar 5 μm, magnification × 1.0 k) (scale bar 2 μm, magnification × 2.0 k) (*n* = 4/group)

VB enhanced the cell viability of Aβ_1-42_-exposed U251 cells at a concentration of 0.25 and 1 μM (*p* < 0.05), but showed no significant effects on cell viability in normal U251 cells (Fig. 2a). VB reduced the apoptosis rate by greater than 10.79% (Fig. 2b), strongly inhibited intracellular Ca^2+^ flux (Fig. 2c), and suppressed the over-accumulation of ROS (Fig. 2d) in Aβ_1-42_-exposed U251 cells. TEM showed that expansion of the perinuclear space, expansion and partial degranulation of rough ER, and increased mitochondrial electron density were visible in Aβ_1-42_-incubated U251 cells, and these effects were partially reversed by VB treatment (Fig. 2e).

### VB improves memory and spatial cognition in APP/PS1 mice due to its suppression of Aβ deposition and tau protein accumulation

**Fig. 3:**
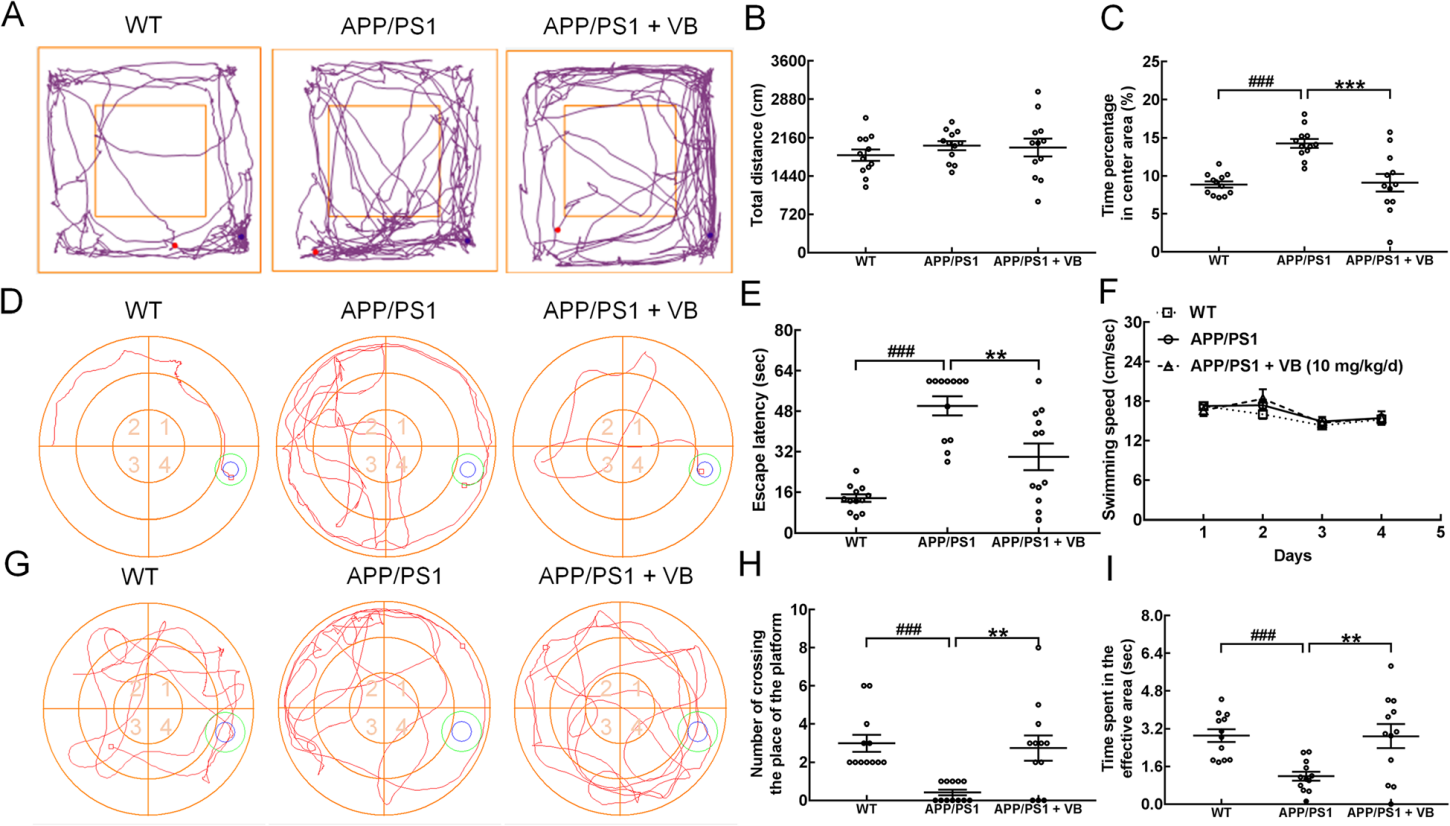
VB improved memory and spatial cognition in APP/PS1 mice. **a** Representative running traces of APP/PS1 mice in different groups in the OFT. The blue dot represents the starting position, and the red dot represents the ending position. The small square represents the central area, and other area of the large square represents the surrounding area. Quantitative analysis of **b** the total distance moved and **c** the time spent in the center area in the OFT. **d** Representative swimming traces of different groups of APP/PS1 mice in the MWM test. The water maze area is defined by four quadrants. The blue circle in quadrant 4 represents the platform position, and the green circle represents the effective area. The small red square represents the end position of the mouse. Quantitative analysis of **e** the time and **f** speed to find the platform in the MWM test. **g** Representative swimming traces of APP/PS1 mice in different groups in the spatial probe test. The blue circle represents the location of the platform during training. Quantitative analysis of **h** the number of times a mouse crossed the previous location of the platform and **i** the time spent in the effective area in the spatial probe test. The data are presented as mean ± SEM (*n* = 12/group). ^###^*p* < 0.001 vs. WT mice, and **p < 0.01, ***p < 0.001 vs. APP/PS1 mice

Healthy mice prefer to remain on the periphery of the open field, whereas mice with cognitive impairment explore the central area [40]. APP/PS1 mice spent more time in the central area than did WT mice (*p* < 0.001). The abnormal behaviors were decreased after VB administration (*p* < 0.001) (Fig. 3a–c). MWM tests are commonly used to study the spatial learning and memory of animals [3]. VB improved spatial cognition, learning, and memory ability, as suggested by the obvious decrease in the escape-latency time of APP/PS1 mice on the 35th day with no significant change in swimming speed in the MWM test (*p* < 0.01) (Fig. 3d–f). This was also shown by the obvious increase in the number of times the VB mice crossed the previous location of the platform, and the increase in the time that mice spent in the effective area on the 36th day (*p* < 0.01) (Fig. 3g–i).

**Fig. 4:**
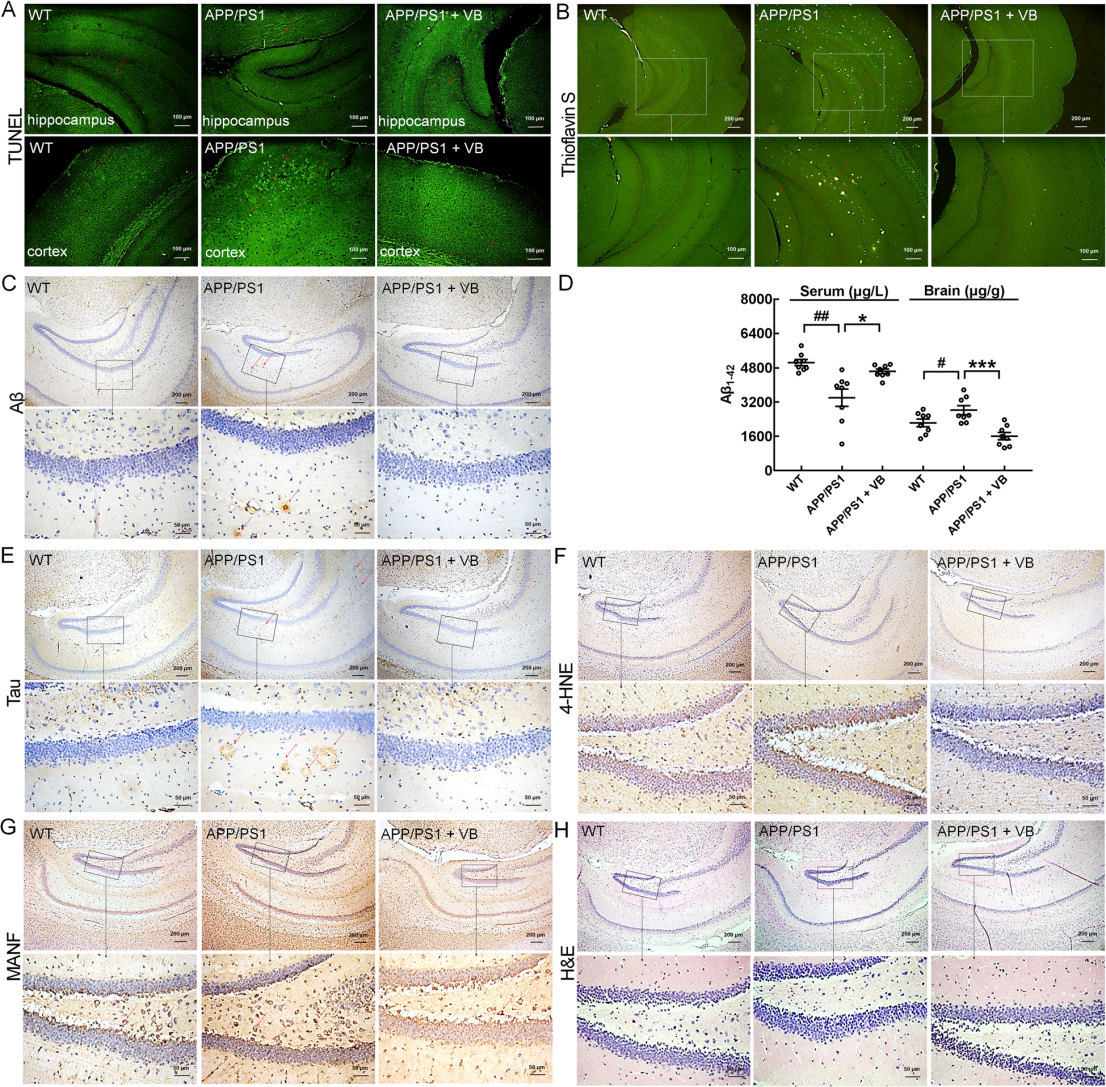
The protection of VB against AD-like symptoms in APP/PS1 mice. **a** TUNEL staining showed that VB reduced neuronal apoptosis in the hippocampus and cortex of APP/PS1 mice. Apoptotic cells are marked with red arrows. **b** VB suppressed the levels of Aβ_1-42_ in the mouse hippocampus on thioflavin-S staining. Aβ plaques are marked with red arrows. **c** VB reduced the expression levels of Aβ_1-42_ in the mouse hippocampus. Aβ plaques are marked with red arrows. **d** Enzyme-linked immunosorbent assay analysis showed that VB reduced the concentrations of Aβ_1-42_ in mouse brains and increased the concentrations of Aβ_1-42_ in sera. The data are presented as mean ± SEM. ^#^*p* < 0.05 and ^##^*p* < 0.01 vs. WT mice, and *p < 0.05 and ***p < 0.001 vs. APP/PS1 mice. **e** VB decreased the high concentrations of phosphorylated tau protein in mouse hippocampus. NFTs are marked with red arrows. **f** VB inhibited the 4-HNE protein expression levels in the mouse hippocampus. 4-HNE is marked with red arrows. **g** VB decreased the concentrations of MANF protein in the mouse hippocampus. MANF is marked with red arrows. **h** VB showed no significant pathologic alteration of the mouse hippocampus via H&E staining. The magnification is × 4 and × 20, and the scale bar is 200 and 50 μm, respectively, for all image pairs except for **a**, where the magnification is × 10 and the scale bar is 100 μm. *n* = 3/group for all of the tests except for **d**, where *n* = 8/group

VB reduced apoptosis in the cortex and hippocampus of APP/PS1 mice (Fig. 4a). Senile plaques formed by the aggregation and deposition of Aβ_1-42_ and NFTs formed by the over-expression of phosphorylated tau protein are the two pathological signs of AD [7]. Compared with vehicle-treated APP/PS1 mice, VB-treated mice had reduced expression levels of Aβ in the hippocampus (Fig. 4b, c), lowered concentrations of Aβ_1-42_ in brain lysates (*p* < 0.001) (Fig. 4d), and increased concentrations of Aβ_1-42_ in serum (*p* < 0.05) (Fig. 4d). VB suppressed the elevated concentrations of phosphorylated tau protein in the brains of APP/PS1 mice (Fig. 4e). 4-HNE, a neurotoxicity product of lipid peroxidation, is highly expressed in the brain and cerebrospinal fluid of AD patients [41]. The concentrations of 4-HNE in the hippocampus of APP/PS1 mice were substantially reduced by VB administration (Fig. 4f). MANF is a key factor in alleviating ER stress and maintaining ER homeostasis [42] and has been confirmed to be upregulated during ER stress and can resist Aβ toxicity by attenuating Aβ-induced ER stress [43]. In the hippocampus of APP/PS1 mice, VB decreased the expression levels of MANF, suggesting that VB protected against ER stress (Fig. 4g).

No significant changes in body weight (Additional file 1: Fig. S2) or pathologic changes in the brain (Fig. 4h), spleen (Additional file 1: Fig. S3A), liver (Additional file 1: Fig. S3B), or kidney (Additional file 1: Fig. S3C) of experimental mice were noted.

### The protective effect of VB against AD is related to its regulation of ER stress

**Fig. 5:**
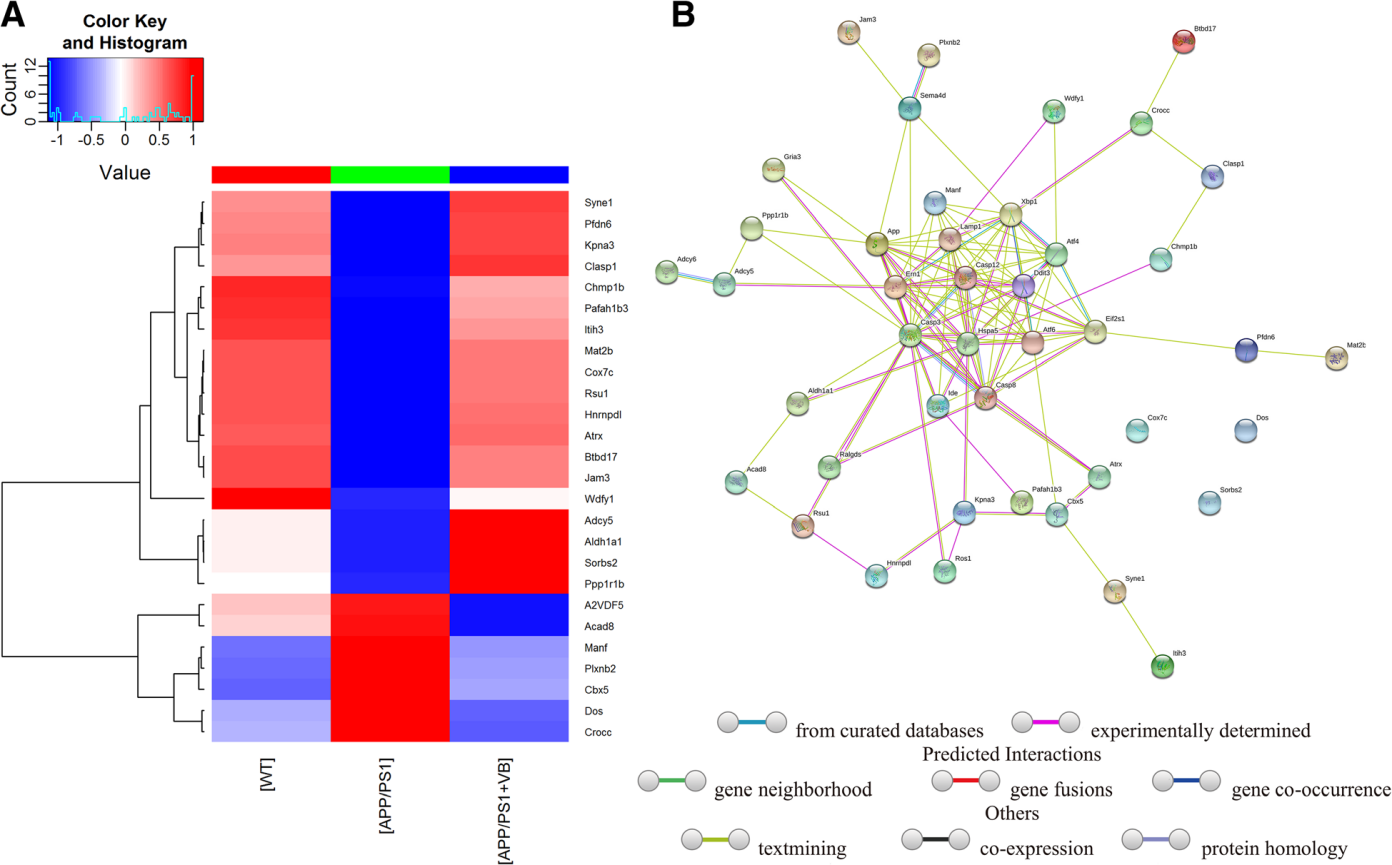
Heatmap visualization and network analysis of differential expression proteins in mouse hippocampus. **a** Proteomics heatmap of WT mice, APP/PS1 mice, and APP/PS1 + VB mice. Red represents high expression levels, and blue represents low expression levels. **b** Protein STRING interaction diagram. The meaning of different colored lines is shown in the figure

LC-MS/MS was used to separate and analyze the peptides in the hippocampus of APP/PS1 mice. The data were processed with MaxQuant and label-free quantification, with matching between run and intensity-based absolute quantification, and proteins with a ratio of A/B > 1.5 or < 0.66 were defined as significantly differentially expressed (where A and B represent the expression level of any protein in two groups). Proteins showing significant differences in expression between APP/PS1 mice and WT mice and between APP/PS1 + VB mice and APP/PS1 mice were used to perform clustering analysis. Compared with non-treated APP/PS1 mice, VB-treated mice had increased concentrations of 19 proteins and decreased concentrations of 7 proteins (Fig. 5a) (Table 2).

**Table 2:** Proteins with significantly different expression levels in proteomics

Number	Gene names	Unique peptides	fc. APP/PS1-WT	fc. APP/PS1 + VB -APP/PS1
**Upregulated proteins by VB (number: 19)**				
1	Syne1	7	0.21	7.12
2	Pfdn6	2	0.17	4.01
3	Kpna3	4	0.10	4.15
4	Clasp1	8	0.02	71.45
5	Chmp1b	2	0.63	1.50
6	Pafah1b3	3	0.14	3.35
7	Itih3	5	0.14	1.83
8	Mat2b	5	0.12	6.65
9	Cox7c	2	0.12	45.81
10	Rsu1	2	0.21	3.93
11	Hnrnpdl	5	0.05	15.48
12	Atrx	3	0.03	32.42
13	Btbd17	8	0.45	2.07
14	Jam3	4	0.14	3.84
15	Wdfy1	6	0.46	1.53
16	Adcy5	13	0.50	3.01
17	Aldh1a1	10	0.72	1.79
18	Sorbs2	8	0.18	7.46
19	Ppp1r1b	9	0.55	2.85
**Downregulated proteins by VB (number: 7)**				
1	A2VDF5	2	1.50	0.12
2	Acad8	5	1.59	0.09
3	Manf	4	5.54	0.26
4	Plxnb2	6	5.55	0.28
5	Cbx5	3	6.23	0.29
6	Dos	3	2.99	0.19
7	Crocc	4	3.64	0.10

fc. APP/PS1-WT: the ratio of protein between saline-treated APP/PS1mice and WT mice; fc. APP/PS1 + VB-APP/PS1: the ratio of protein between VB-treated APP/PS1mice and saline-treated APP/PS1mice

STRING is a database of known and predicted protein-protein interactions. In this study, the interactions of 43 proteins among experimental groups were analyzed. Protein-protein interaction results showed that VB had a significant effect on ER-related factors (Fig. 5b).

**Fig. 6:**
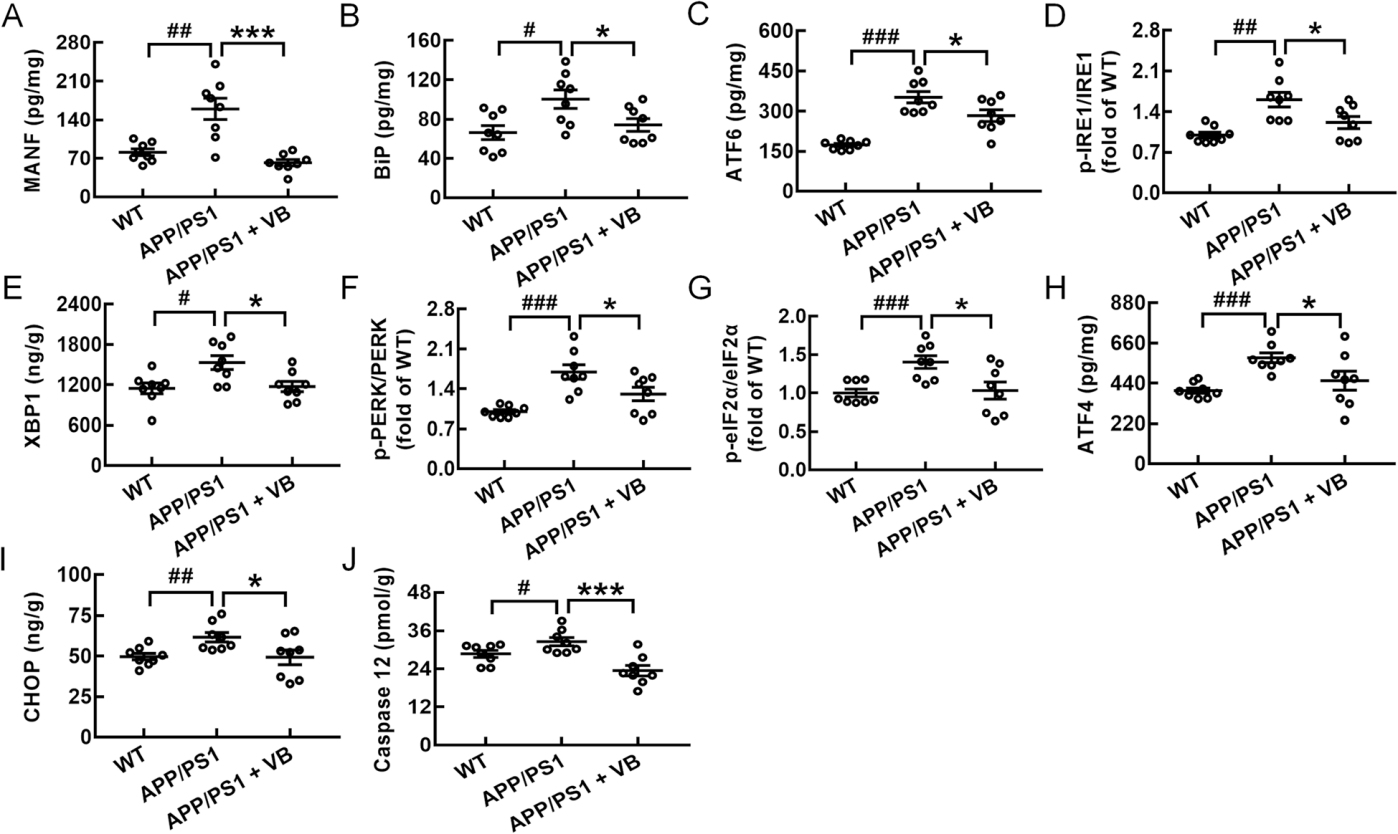
VB protected APP/PS1 mice against ER stress. VB reduced the concentrations of the following proteins related to ER stress in mice brains by ELISA: **a** MANF, **b** BiP, **c** ATF6, **d** p-IRE1/IRE1, **e** XBP1, **f** p-PERK/PERK, **g** p-eIF2α/eIF2α, **h** ATF4, **i** CHOP, and **j** caspase 12. The data are presented as mean ± SEM (*n* = 8/group). ^#^*p* < 0.05, ^##^*p* < 0.01, and ^###^*p* < 0.001 vs. WT mice, *p < 0.05 and ***p < 0.001 vs. APP/PS1 mice

Excessive accumulation of Aβ and tau protein can cause abnormal ER stress, which in turn promotes AD [5]. According to proteomic screening and analysis, 10 proteins related to ER stress were further confirmed in this experiment via ELISA. Encouragingly, VB was effective in alleviating ER stress, as suggested by its reduction in the concentrations of MANF (an ER resident protein that regulates ER homeostasis in neurons [44]) (*p* < 0.001) (Fig. 6a), BiP (an ER chaperone that regulates the homeostasis of protein folding [45]) (*p* < 0.05) (Fig. 6b), ATF6 (an ER transmembrane receptor related to ER stress [46]) (*p* < 0.05) (Fig. 6c), p-IRE1/IRE1 (an ER-located kinase that mediates both adaptive and proapoptotic programs under ER stress [47]) (*p* < 0.05) (Fig. 6d), XBP1 (a transcription factor that activates the UPR target gene cluster [47]) (*p* < 0.05) (Fig. 6e), phosphorylated protein kinase-like endoplasmic reticulum kinase (p-PERK)/PERK (a type I transmembrane kinase that is positioned at the ER membrane to regulate ER stress [5]) (*p* < 0.05) (Fig. 6f), p-eIF2α/eIF2α (a kinase related to cognitive disorders and memory impairment due to its over-activation [48]) (*p* < 0.05) (Fig. 6g), ATF4 (a transcription factor that controls protein folding and redox homeostasis in the ER [5]) (*p* < 0.05) (Fig. 6h), CHOP (an important mediator of ER stress-induced cell arrest and apoptosis [49]) (*p* < 0.05) (Fig. 6i), and caspase12 (an ER resident pro-caspase that is activated under ER stress [50]) (*p* < 0.001) (Fig. 6j) in the brains of APP/PS1 mice.

Proteins related to apoptosis and oxidative stress were also detected. Compared with vehicle-treated APP/PS1 mice, VB-treated mice had reduced concentrations of caspase 3 (which promotes the release of cytochrome c from mitochondria and the collapse of membrane potential [51]) (*p* < 0.001) (Additional file 1: Fig. S4A), caspase 8 (which initiates the apoptotic pathway in mitochondria [52]) (*p* < 0.001) (Additional file 1: Fig. S4B) and ROS (which are species that are directly responsible for oxidative stress [53]) (*p* < 0.001) (Additional file 1: Fig. S4C), and increased the concentrations of IDE (an important enzyme responsible for the degradation and clearance of Aβ [54]) (*p* < 0.05) (Additional file 1: Fig. S4D) in the brains of APP/PS1 mice. These findings support the protective effects of VB against apoptosis in AD.

### VB regulated the expressions of protein related to ER stress

**Fig. 7:**
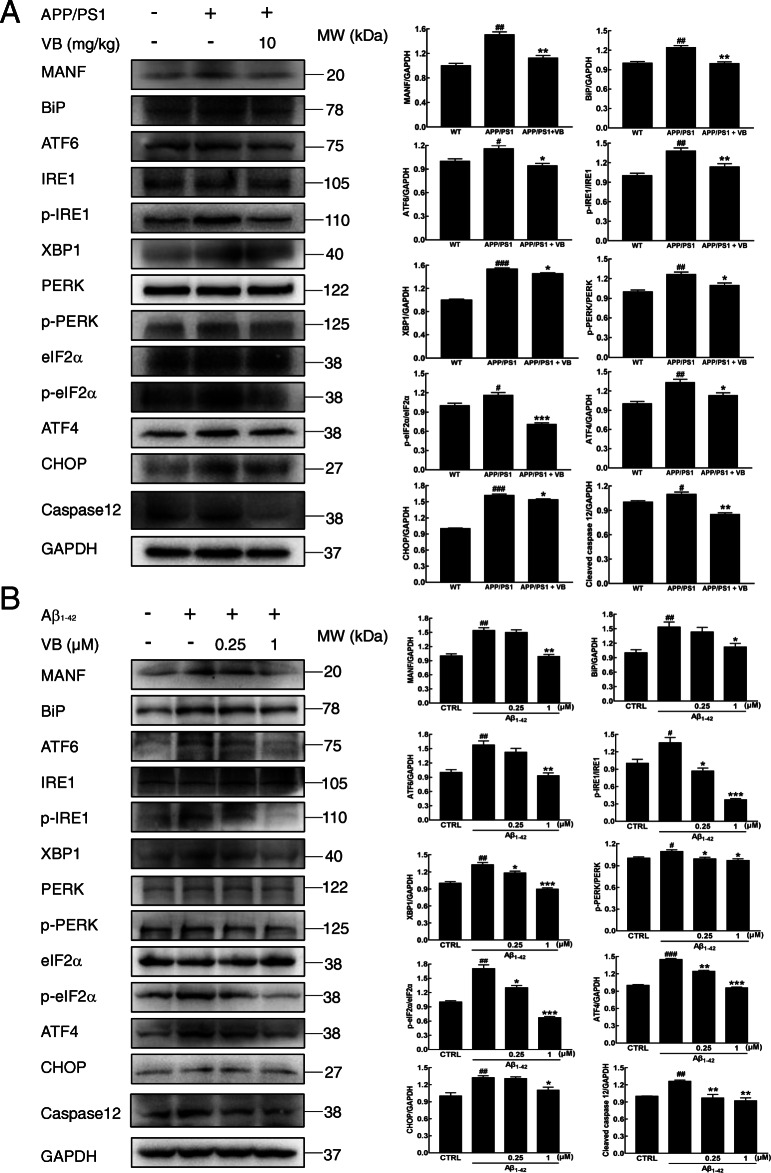
VB attenuated ER stress in APP/PS1 mice and Aβ_1-42_-exposed U251 cells. VB reduced the expression levels of MANF, BiP, ATF6, XBP1s, ATF4, CHOP, and caspase 12 and the levels of phosphorylated IRE1, PERK, and eIF2α in **a** the brain lysates of APP/PS1 mice and **b** Aβ_1-42_-exposed U251 cells. GAPDH was used as a loading control and for band-density normalization. Phosphorylated proteins were normalized using the related total protein concentration. The data are presented as means ± SEM (*n* = 4/group). ^#^*p* < 0.05, ^##^*p* < 0.01, and ^###^*p* < 0.001 vs. WT mice or non-treated cells and *p < 0.05, **p < 0.01, and ***p < 0.001 vs. APP/PS1 mice or Aβ_1-42_-exposed cells

In the brain lysates of APP/PS1 mice and Aβ_1-42_-exposed U251 cells, VB reduced the concentrations of MANF (*p* < 0.01), BiP (*p* < 0.05), ATF6 (*p* < 0.05), p-IRE1/IRE1 (*p* < 0.05), XBP1 (*p* < 0.05), p-PERK/PERK (*p* < 0.05), p-eIF2α/eIF2α (*p* < 0.05), ATF4 (*p* < 0.05), CHOP (*p* < 0.05), and Caspase12 (*p* < 0.01) (Fig. 7a, b).

## Discussion

In the present study, we found VB attenuates AD symptoms and pathology and decreases ER stress in mice models and cultured cells. These findings, for the first time, prove VB inhibits the progression of AD via modulating ER stress in the central nervous system, suggesting VB is a potential therapeutic medicine in the future. These findings also support the hypothesis that ER stress mediates the progression of AD [23].

Here, we demonstrated that in Aβ_1-42_-damaged U251 cells, VB increased viability and reduced the concentrations of intracellular Ca^2+^ and ROS in vitro. In the early stages of AD, mitochondrial damage causes intracellular ROS accumulation [55], eventually leading to mitochondrial dysfunction [56]. This promotes Aβ accumulation [57] and induces ER stress leading to hyper phosphorylation of tau protein [26, 43]. Aβ_1-42_ treatment has been reported to change the morphology of astrocytes and regulates the release of interleukin-1 beta and tumor necrosis factor alpha by activating PERK/elF2α-dependent ER stress, which induces neuroinflammation and apoptosis [27]. Notably, VB significantly improved the morphological characteristics of mitochondria and ER in U251 cells. These data are consistent with previous reports [16, 22, 58] and confirmed the neuroprotective activity of VB in vitro.

As the commonly used mouse model of AD, the APP/PS1 double-transgenic mouse displays over-expression of amyloid precursor protein (APP) and presenilin 1 and decreasing memory ability with age [59]. In the 9-month-old APP/PS1 mouse, VB reduced anxiety, improved memory and spatial cognition, suppressed the deposition of Aβ and tau protein phosphorylation, and decreased the overexpression of 4-HNE and MANF in the hippocampus. Excessive accumulation of Aβ and increased phosphorylation of tau protein can trigger ER stress [5]. Subsequently, continuous ER stress induces hyperphosphorylation of tau, which initiates a vicious cycle and aggravates the AD process [26]. Furthermore, ER stress and over-accumulation of ROS in the brain promote lipid peroxidation, generating 4-HNE, which inhibits dephosphorylation of tau [60]. Hyperphosphorylated tau shows much greater deleterious effects (via the formation of NFTs) on cognitive function than does Aβ deposition, and as reported, knock-out of tau can inhibit Aβ-induced memory and cognitive impairment [61, 62]. In this study, VB helped to improve the memory and cognitive ability of APP/PS1 mice.

Label-free quantification proteomics analysis was performed to screen for significantly differentially expressed proteins, and their interactions. Consistent with our findings, among the 26 proteins significantly influenced by VB, we identified MANF, an ER stress response protein. As a member of the fourth family of neurotrophic factors, MANF has been proven to protect and repair dopaminergic neurons and maintain the homeostasis of ER proteins [42, 43]. According to our STRING protein interaction mapping analysis, MANF is found to interact with ATF4, XBP1, and ATF6. Knockout of MANF upregulates the expression of ATF4, XBP1, and ATF6 in cells [43]. As a transcription factor, ATF4 is responsible for controlling protein homeostasis in the ER [5], XBP1 activates the UPR target [47], and ATF6 targets UPR-related proteins after activation [23]. Under chronic or irreversible ER stress, UPR activation promotes the release of Ca^2+^ in the ER and activates the intrinsic and extrinsic apoptotic pathways by regulating the B cell lymphoma 2 (Bcl-2) and caspase protein families [63]. Based on the results of TEM of U251 cells and label-free quantification proteomics analysis, we speculate that VB improves the pathological condition of AD mice, possibly due to its regulation of ER stress.

Furthermore, ELISA and western blot analysis were performed to detect the concentrations of related proteins screened by label-free quantitative proteomics analysis in APP/PS1 mice brains and Aβ_1-42_-exposed U251 cells. Surprisingly, these proteins belong to the three branches of UPR signaling: PERK/eIF2α/ATF4, IRE1/XBP1, and ATF6. According to previous reports, ATF4 is a downstream target of PERK/eIF2α. Activated PERK promotes the phosphorylation of eIF2α, which further improves the efficiency of translation of ATF4. CHOP is activated by ATF4 and triggers cell death programs by regulating Bcl-2, AKT, and JNK [5, 24]. XBP1 is a downstream target of IRE1. IRE1 is auto-phosphorylated after dissociating from BiP and promotes the unconventional digestion of XBP1 mRNA into XBP1s [24], which mediates apoptosis by increasing the activation of caspases [64]. In the case of ER stress, ATF6 is separated from BiP and translocated to the Golgi apparatus, where it is lysed by the proteases SP1 and SP2 to generate ATF6f, which induces cell apoptosis [5, 43]. In this study, we found that VB inhibited all three of the branches of the UPR signal, thereby preventing ER stress and, in turn, delaying the progress of AD. Our data suggest the important roles of VB-mediated protection during ER stress against AD-like symptoms.

In summary, we found that VB relieved ER stress by inhibiting the UPR signal and enhancing mitochondrial and ER morphology in Aβ_1-42_-exposed U251 cells. Simultaneously, VB relieved the pathological condition of APP/PS1 mice, thereby improving their memory and cognitive ability. However, the specific target and binding sites of VB are still unknown. Furthermore, how VB prevents apoptosis by improving ER stress should be verified in future research. Recently, it has been reported that ER stress can cause inflammation and that this leads to neuronal apoptosis and synaptic loss [65]. In future research, we will therefore explore the molecular mechanism of the neuroprotective effects of VB in greater depth. Finally, we did not measure how much VB passes through the blood brain barrier, so we do not know whether VB directly targets brain cells or reduces systemic ER stress and indirectly attenuate neuronal ER stress.

## Conclusions

To the best of our knowledge, this is the first study to report that the neuroprotective effects of VB against AD in APP/PS1 mice and Aβ_1-42_-exposed U251 cells occurs via VB increasing resistance to ER stress. Our findings support the role of VB as a candidate agent for further research as an AD remedy, which also support the hypothesis that ER stress mediates the progression of AD [23]. Further mechanism study is needed to illustrate the molecular mechanism of VB and the contribution of ER stress in the progression of AD.

## Supplementary information

**Additional file 1: Figure S1.** The structure of verbascoside (Cas.NO 61276-17-3). **Figure S2** The changes on body weight of APP/PS1 mice during the six-week experimental period. (*n* = 12). *p* > 0.05 vs. WT mice, and *p* > 0.05 vs. APP/PS1 mice. Data are the mean ± SEM. **Figure S3** VB showed no significant pathologic alternations on **(A)** spleen, **(B)** liver and **(C)** kidneys of mice via H & E staining (Magnification ×20, Scale bar: 50 μm) (*n* = 3). **Figure S4** VB reduced the levels of **(A)** caspase 3, **(B)** caspase 8 and **(C)** ROS, and increased **(D)** IDE in brain of APP/PS1 mice analyzing via ELISA (*n* = 8). ^#^*p*< 0 .05 and ^##^*p* < 0.01 vs. WT mice, and *p < 0.05 and ***p < 0.001 vs. APP/PS1 mice. Data are the mean ± SEM.

## Data Availability

The datasets used and/or analyzed during the current study are available from the corresponding author on reasonable request.

## Abbreviations

AD: Alzheimer’s disease
ATF4: Activating transcription factor-4
ATF6: Activating transcription factor-6
APP/PS1: APPswe/PSEN1dE9 transgenic
ARRIVE: Animal Research: Reporting In Vivo Experiments
Aβ: Amyloid β
Bcl-2: B cell lymphoma 2
BiP: Immunoglobulin-binding protein
CHOP: C/EBP-homologous protein
eIF2α: Eukaryotic initiation factor 2
ELISA: Enzyme-linked immunosorbent assay
ER: Endoplasmic reticulum
H&E: Hematoxylin and eosin
IDE: Insulin-degrading enzyme
IRE1: Inositol-requiring protein 1
MANF: Mesencephalic astrocyte-derived neurotrophic factor
MWM: Morris water maze
NFTs: Neurofibrillary tangles
OFT: Open-field test
PERK: Pancreatic ER kinase
PMSF: Phenylmethanesulfonyl fluoride
RIPA: Radio-immunoprecipitation assay
ROS: Reactive oxygen species
SEM: Means ± standard errors of the mean
TEM: Transmission electron microscopy
TUNEL: Terminal deoxynucleotidyl transferase-mediated deoxyuridine triphosphate nick end labeling
UPR: Unfolded protein response
U251: Human glioma
VB: Verbascoside
WT: Wild-type
XBP1: X-box binding protein 1
4-HNE: 4-Hydroxynonenal
