# Supplementary material for: Neuroprotective effects of verbascoside against Alzheimer’s disease via the relief of endoplasmic reticulum stress in Aβ-exposed U251 cells and APP/PS1 mice
Source: J Neuroinflammation. 2020 Oct 18;17:309. doi: 10.1186/s12974-020-01976-1 (PMC7570123; doi:10.1186/s12974-020-01976-1)
Supplement: Supplementary file 1 — Additional file 1: Figure S1. The structure of verbascoside (Cas.NO 61276-17-3). Figure S2 The changes on body weight of APP/PS1 mice during the six-week experimental period. (n = 12). p > 0.05 vs. WT mice, and p > 0.05 vs. APP/PS1 mice. Data are the mean ± SEM. Figure S3 VB showed no significant pathologic alternations on (A) spleen, (B) liver and (C) kidneys of mice via H & E staining (Magnification ×20, Scale bar: 50 μm) (n = 3). Figure S4 VB reduced the levels of (A) caspase 3, (B) caspase 8 and (C) ROS, and increased (D) IDE in brain of APP/PS1 mice analyzing via ELISA (n = 8). #p< 0 .05 and ##p < 0.01 vs. WT mice, and *p < 0.05 and ***p < 0.001 vs. APP/PS1 mice. Data are the mean ± SEM. [file 12974_2020_1976_MOESM1_ESM.docx]

**Supplemental Figure List**

**

**

**Figure S1** The structure of verbascoside (Cas.NO 61276-17-3).





**Figure S2** The changes on body weight of APP/PS1 mice during the six-week experimental period. (n=12). *p* > 0.05 vs. WT mice, and *p* > 0.05 vs. APP/PS1 mice. Data are the mean ± SEM.


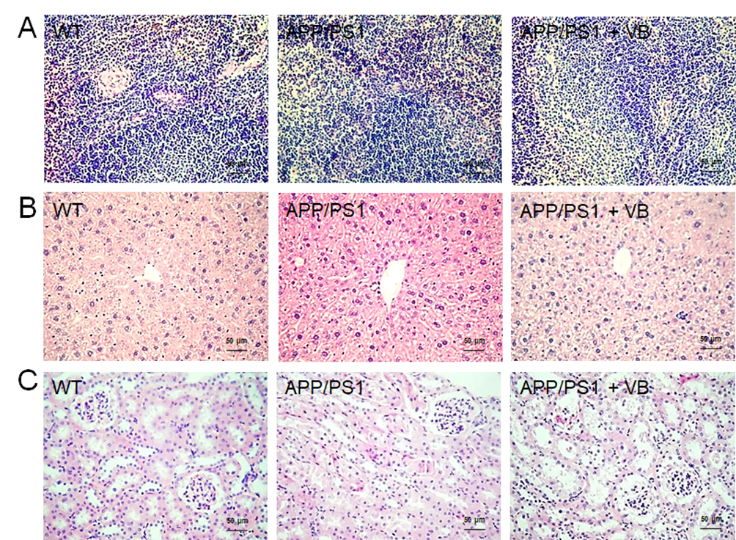


**Figure S3** VB showed no significant pathologic alternations on **(A)** spleen, **(B)** liver and **(C)** kidneys of mice via H & E staining (Magnification ×20, Scale bar: 50 μm) (n=3).





**Figure S4** VB reduced the levels of **(A)** caspase 3, **(B)** caspase 8 and **(C)** ROS, and increased **(D)** IDE in brain of APP/PS1 mice analyzing via ELISA (n=8). ^#^*p* < 0.05 and ^##^*p* < 0.01 vs. WT mice, and **p* < 0.05 and ****p* < 0.001 vs. APP/PS1 mice. Data are the mean ± SEM.
